# *hCD2-iCre* and *Vav-iCre* Mediated Gene Recombination Patterns in Murine Hematopoietic Cells

**DOI:** 10.1371/journal.pone.0124661

**Published:** 2015-04-17

**Authors:** Sabine Siegemund, Jovan Shepherd, Changchun Xiao, Karsten Sauer

**Affiliations:** 1 Department of Immunology and Microbial Science, The Scripps Research Institute, La Jolla, California, United States of America; 2 Department of Cell and Molecular Biology, The Scripps Research Institute, La Jolla, California, United States of America; Emory University, UNITED STATES

## Abstract

Cre-recombinase mediated conditional deletion of *Lox-P* site flanked ("*floxed*") genes is widely used for functional gene annotation in mice. Many different *Cre*-transgenic mouse lines have been developed for cell-type specific gene disruption. But often, the precise tissue-patterns of *Cre* activity remain incompletely characterized. Two widely used transgenes for conditional gene recombination in hematopoietic cells are *Vav-iCre* driven from the *murine Vav1* promotor, and *hCD2-iCre* driven from the *human CD2* promotor. *Vav-iCre* expresses active Cre in fetal and adult hematopoietic stem cells and all descendants, *hCD2-iCre* in immature and mature B and T lymphocytes. To better characterize which hematopoietic cells contain *hCD2-iCre* activity, we compared EYFP fluorescence in *hCD2-iCre^+/-^ R26-stop-EYFP^+/- ^*and *Vav-iCre^+/- ^R26-stop-EYFP^+/-^*mice. *R26-stop-EYFP *ubiquitously encodes *EYFP *preceded by a *floxed stop* cassette. By removing it, Cre activity induces measurable EYFP expression. Our results confirm the known activity patterns for both *Cre* transgenes and unveil additional *hCD2-iCre* mediated reporter gene recombination in common lymphoid progenitors, in natural killer cells and their progenitors, and in plasmacytoid and conventional dendritic cells. This supports previously proposed common lymphoid origins for natural killer cells and subsets of dendritic cells, and indicates the need to consider pleiotropic effects when studying *hCD2-iCre* mediated conditional knockout mice. *Vav-iCre^+/- ^R26-stop-EYFP^+/-^*mice did not show the non-hematopoietic recombination in vascular endothelial cells seen in other *Vav-Cre* mouse lines, but displayed an unexpected *Vav-iCre* mediated recombination in a bone cell subset lacking hematopoietic markers. This pinpoints the need to consider stromal cell contributions to phenotypes of *Vav-iCre* mediated conditional knockout mice. Altogether, our data provide the first detailed assessment of *hCD2-iCre* and *Vav-iCre* mediated deletion of *floxed* genes during lymphocyte development from hematopoietic stem cells and open up novel applications for either *Cre-transgenic* mouse line.

## Introduction

Cre-recombinase mediated conditional deletion of *Lox-P* site flanked ("*floxed*") genes is widely used for functional gene annotation in mice. Many different *Cre*-transgenes are available for cell-type specific or drug-induced disruption of a targeted gene [1,2]. Transgenes encoding *improved Cre* (*iCre*) optimize Cre expression through optimized codon usage, removed putative cryptic splice sites and reduced CpG content to limit epigenetic silencing [3]. However, transgenic Cre activity in untargeted cell types can compromise the desired cell type specificity of Cre mediated gene recombination [4]. For example, *Vav1* promoter driven Cre (*Vav-Cre)* or improved Cre (*Vav-iCre*) disrupt *floxed* genes in fetal and adult hematopoietic stem cells (HSC) and their descendants, which ultimately form all blood cells. Thus, *Vav-Cre* is commonly used for pan-hematopoietic gene disruption [2,3,5–9]. But certain *Vav-Cre* lines also delete in vascular endothelial cells (EC) or in precursors which do not express Vav, possibly due to *Cre-transgene* or *LacZ reporter* insertion effects [6]. This might indirectly affect hematopoiesis, in particular HSC quiescence in bone marrow (BM) vascular endothelial niches [10]. *Vav-iCre* transgene expression also occurs in the testis [3]. Thus, for many Cre-lines a better characterization of the precise tissue-patterns of *Cre* activity is required before observed phenotypes can be unambiguously linked to the specific disruption of the targeted gene in the targeted cell type.

Cell type-specific Cre activity can be visualized in *R26-stop-EYFP* mice where a *EYFP* gene preceded by a *floxed stop* cassette was knocked into the ubiquitously expressed *ROSA26* locus. Stop removal by Cre recombination induces measurable EYFP expression in those cells harboring active Cre without significant leaky EYFP expression [11]. Flow cytometry can quantify both the proportion of EYFP expressing cells, and the extent of Cre activity in these which correlates with the EYFP mean fluorescence intensity (MFI). Once the *Stop* cassette has been removed, EYFP expression continues even in the absence of Cre. This enables cell tracing and fate mapping studies, but can also mask Cre-inactivation after a Cre-expressing developmental or physiological stage.

Mice transgenic for *iCre* under control of the *human CD2* promoter (*hCD2-iCre*) are a popular tool to conditionally delete genes in immature thymocytes, mature T cells and B cells without reported effects on myeloid cells [3,12,13]. The precise stage of hematopoietic development where *hCD2-iCre* is first active, and to what extent *hCD2-iCre* also deletes *floxed* genes in other hematopoietic cells is unknown. Testis expression shows that *hCD2-iCre* can be expressed in non-hematopoietic cells [3]. To better characterize the activity patterns of this important *Cre*-line, we analyzed EYFP expression in hematopoietic cell and progenitor subsets of *hCD2-iCre* ^*+/-*^ *R26-stop-EYFP* ^*+/-*^ mice and controls. For comparison, we also analyzed *Vav-iCre* ^*+/-*^ *R26-stop-EYFP* ^*+/-*^ mice and controls.

Our results confirm the known activity patterns for both *Cre* transgenes and unveil additional *hCD2-iCre* mediated reporter gene recombination in common lymphoid progenitors (CLP), NK cell progenitors (NKP), NK cells, all pDC and ~20% of conventional dendritic cells (cDC). This is consistent with the proposed CLP origin of NK cells and of subsets of pDC and cDC [14–18], and shows the utility of *hCD2-iCre* for conditional gene disruption in these cell types. Unexpected *Vav-iCre* activity in bone cells which do not express hematopoietic surface markers pinpoints a need to consider stromal or niche cell contributions to phenotypes of *Vav-iCre* mediated conditional knockout mice.

## Materials and Methods

### Ethics statement

This study was carried out in strict accordance with the recommendations in the Guide for the Care and Use of Laboratory Animals of the National Institutes of Health. The protocol was approved by the Institutional Animal Care and Use Committee (IACUC, Assurance Number: A3194-01) of The Scripps Research Institute (TSRI). All efforts were made to minimize animal suffering. Mice were euthanized by CO_2_/O_2_ mixture or halothane volatile anesthetic overdose inhalation.

### Mice

*hCD2-iCre* and V*av-iCre* transgenic mice [3] were obtained from The Jackson Laboratory [*B6*.*Cg-Tg(CD2-cre)4Kio/J*, stock no. 008520, and *B6*.*Cg-Tg(Vav1-cre)A2Kio/J*, stock no. 008610]. Both lines were independently bred to *R26-stop-EYFP* mice [11] [*B6*.*129X1-Gt(ROSA)26Sortm1(EYFP)Cos/J*, The Jackson Laboratory, order no. 006148] and genotyped by PCR as described in [3,11] or on the Jackson Laboratory webpage. *hCD2-iCre* is homozygous lethal [13]. Thus, *hCD2-iCre* ^+/-^ *R26-stop-EYFP* ^*+/-*^, V*av-iCre* ^*+/-*^ *R26-stop-EYFP* ^*+/-*^, and *R26-stop-EYFP* ^*+/-*^ mice were used for analysis. EYFP expression in thymocytes was determined in 5.5–7.5 week old mice, as the thymus starts to undergo atrophy in older mice. Otherwise, EYFP expression was determined in 6–17 week old mice. All mice were housed in the TSRI Specific Pathogen Free (SPF) facility with a 12 hr light cycle, and were given food and water *ad libitum*.

### Cell preparation

BM, thymocyte and splenocyte single cell suspensions were prepared and BM and spleen red blood cells lysed with BD Pharmlyse (BD Biosciences) as previously described [19–21]. For isolation of bone cells, whole bones were cleaned from muscle tissue, BM was flushed out, and the bones were then chipped into little pieces and digested with collagenase as described elsewhere [22].

### FACS analyses

Published surface marker staining patterns were used for the analysis of HSC [23], hematopoietic progenitor cell (HPC) subsets [24], common lymphoid progenitors (CLP) [25], endothelial cells (EC), osteoblasts (OB) and mesenchymal stem cells (MSC) [22], B cell [26], T cell [27] and NK cell precursors [21]. If not noted otherwise, all antibodies were purchased from eBiosciences, Biolegend or BD Biosciences. Unspecific antibody (AB) binding was prevented by pre-incubation with anti-CD16/32 AB (clone 93, 1:10 diluted) or, when staining for CD16/32, with 5% rat serum (Stemcell Technologies, cat. no. 19700). The cells were first stained with a Lineage cocktail (Lin) containing biotinylated AB (diluted 1:150 unless noted otherwise) against CD3ε (clone 145-2C11), CD4 (clone GK1.5), CD8α (clone 53–6.7), CD19 (clone 6D5), B220 (clone RA3-6B2), CD11b (clone M1/70), CD11c (clone N418), CD49b (clone DX5), Gr-1 (clone RB6-8C5), TER-119 (clone TER-119) and, when indicated, IL-7Rα (clone A7R34, diluted 1:75) in FACS staining buffer (PBS/3% FCS/0.1% NaN_3_), washed, further stained with anti-mouse c-kit PE-Cy7 (clone 2B8, diluted 1:120), anti-mouse Flk-2 PE (clone A2F10, diluted 1:120), anti-mouse Sca-1 APC-Cy7 (clone D7, diluted 1:120), anti-mouse CD34 Alexa Fluor 700 (clone RAM34, diluted 1:30, eBioscience), anti-mouse CD48 Pacific Blue (clone HM48-1, diluted 1:120), anti-mouse CD150 PerCP-Cy5.5 (clone TC15-12F12.2, diluted 1:150), anti-mouse IL-7Rα Brilliant Violet 421 (clone A7R34, diluted 1:40, Biolegend), anti-mouse CD16/32 APC (clone 93, diluted 1:60), anti-mouse CD45.2 Pacific Blue (clone 104, diluted 1:100), anti-mouse CD31 APC (clone 390, diluted 1:60), anti-mouse CD51 PE (clone RMV-7, diluted 1:60), and APC-Cy7- (diluted 1:120) or Qdot 605- (Life Technologies, cat. no. Q10101MP, diluted 1:120) conjugated streptavidin (SA). Mature hematopoietic cells were stained with anti-mouse CD11c APC (clone 418, diluted 1:12), anti-mouse CD11b Alexa Fluor 700 (clone M1/70, diluted 1:40), anti-mouse Gr-1 PE (clone RB6-8C5, diluted 1:40), anti-mouse F4/80 PerCP-Cy5.5 (clone BM8, diluted 1:40), anti-mouse NK1.1 PE-Cy7 (clone PK136, diluted 1:40), anti-mouse CD3ε APC-eFluor780 (clone 145-2C11, diluted 1:12, eBioscience), anti-mouse B220 Brilliant Violet 421 (clone RA3-6B2, diluted 1:40, Biolegend). B cell precursors were stained with anti-mouse B220 APC-Cy7 (clone RA3-6B2, diluted 1:60), anti-mouse CD43 biotin (clone S7, diluted 1:300, BD Biosciences), anti-mouse BP-1 PE (clone 6C3, diluted 1:60), anti-mouse CD24 APC (clone M1/69, diluted 1:300), anti-mouse IgM PE-Cy7 (clone RMM-1, diluted 1:60), anti-mouse IgD (clone 11-26c.2a, diluted 1:60) and SA-PerCP (diluted 1:120). T cell precursors were first stained with a Lin cocktail containing biotinylated AB (all 1:300 diluted) against CD19 (clone 6D5), B220 (clone RA3-6B2), CD11b (clone M1/70), CD11c (clone N418), CD49b (clone DX5), Gr-1 (clone RB6-8C5), TER-119 (clone TER-119) followed by staining with anti-mouse CD4 Alexa Fluor 700 (clone RM4-5, diluted 1:150, Biolegend), anti-mouse CD8α PE (clone 53–6.7, diluted 1:150), anti-mouse CD44 APC (clone 1M7, diluted 1:600), anti-mouse CD25 PerCP-Cy5.5 (clone PC61, diluted 1:600), anti-mouse CD24 Pacific Blue (clone M1/69, diluted 1:300), anti-mouse c-kit PE-Cy7 (clone 2B8, diluted 1:120), and SA-APC-Cy7 (diluted 1:120). NK cells were stained with anti-mouse CD3ε APC-eFluor780 (clone 145-2C11, diluted 1:200), anti-mouse CD122 eFluor 450 (clone TM-β1, diluted 1:60), anti-mouse NK1.1 PE-Cy7 (clone PK136, diluted 1:60), anti-mouse NKG2D APC (clone CX5, diluted 1:60) and anti-mouse CD11b Alexa Fluor 700 (clone M1/70, diluted 1:300), and with biotinylated AB (all diluted 1:600) against the lineage markers TER-119 (clone TER-119), CD19 (clone 6D5) and Gr-1 (clone RB6-8C5) followed by SA-PerCP (diluted 1:120) stain. All cells were fixed in 1.35% paraformaldehyde/PBS after staining and analyzed within 24 hr. EYFP was detected in the FITC channel. Samples were run on a Beckton Dickinson LSR-II flow cytometer and analyzed with FlowJo software (Treestar).

## Results

### *hCD2-iCre* activity in mature PBL

**Fig 1:**
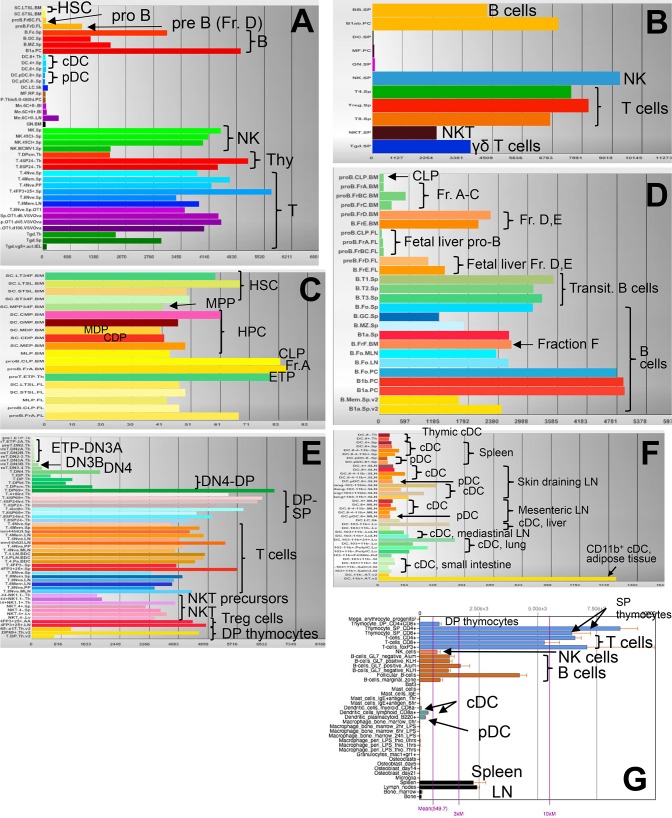
*Murine CD2* mRNA tissue expression profiles. Shown are ImmGen Consortium [53] (www.immgen.org) probe set 10500677 **(A-F)** and BioGPS [54,55] (www.biogps.org) probe set 1418770_at **(G)** *murine CD2* mRNA expression profiles across (A, B) key hematopoietic cell populations, (C) HSC and HPC populations, (D) B cell developmental and mature populations, (E) T cell and NKT cell developmental and mature populations, (F) DC subsets and (G) multiple hematopoietic tissues and cell types. In (G), non-hematopoietic tissues are not shown because they did not express CD2. HSC, hematopoietic stem cells; HPC, hematopoietic progenitor cells; MPP, multipotent progenitors; MDP, monocyte-DC precursors; CDP, common DC precursors; CLP, common lymphoid progenitors; Fr. A, fraction A pre-pro B cells; pro B, pro B cells; Fr. B,C, fraction B and C pro- and early pre B cells; pre B (D), fraction D late pre B cells; Fr. F, fraction F recirculating mature B cells [19,34]; NK, NK cells; Thy, thymocyte; ETP, early thymocyte progenitor; DN3A, DN3B, DN4, CD4^-^CD8^-^ thymocyte subsets; DP, CD4^+^CD8^+^ thymocytes; SP, CD4^+^ and CD8^+^ thymocytes; T_reg_ cells, regulatory T cells; transit. B, transitional B cells.

**Fig 2:**
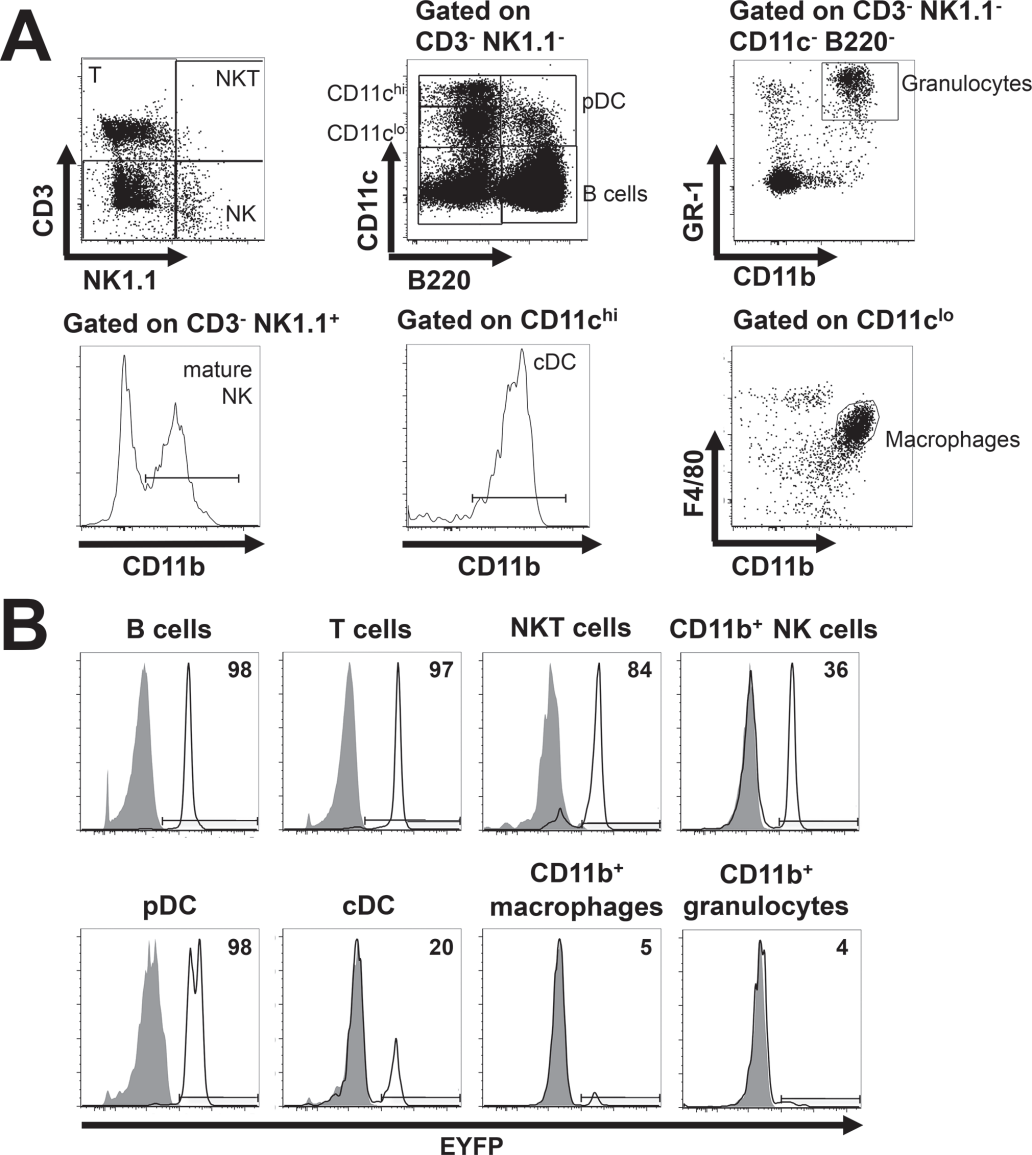
*hCD2-iCre* activity in mature leukocytes. **(A)** Gating strategy. Splenocytes were stained with the indicated AB and analyzed by FACS, gating on CD3^+^NK1.1^-^ T cells, CD3^-^NK1.1^+^CD11b^+^ mature NK cells, CD3^+^NK1.1^+^ NKT cells, CD3^-^ NK1.1^-^CD11c^-^CD11b^+^B220^-^Gr-1^hi^ granulocytes, CD3^-^NK1.1^-^CD11c^lo^B220^+^ pDC, CD3^-^NK1.1^-^CD11c^hi^ CD11b^+^B220^-^ cDC, CD3^-^NK1.1^-^B220^+^ B cells and CD3^-^NK1.1^-^CD11c^lo^F4/80^+^CD11b^+^ macrophages. **(B)** EYFP expression in the indicated mature leukocyte populations from *hCD2-iCre* ^*+/-*^ *R26-stop-EYFP* ^*+/-*^ (open histograms) and *R26-stop-EYFP* ^*+/-*^ mice (shaded histograms). Numbers depict % EYFP^+^ cells in the *hCD2-iCre* ^*+/-*^ *R26-stop-EYFP* ^*+/-*^ mice. Representative of three independent experiments (n = 3 for each genotype).

We used flow cytometry to analyze EYFP expression as a measure of Cre activity in splenic mature leukocytes from *hCD2-iCre* ^*+/-*^ *R26-stop-EYFP* ^*+/-*^ versus *R26-stop-EYFP* ^*+/-*^ mice. Consistent with the initial characterization of the *hCD2-iCre* mice and primarily lymphoid *CD2* expression [3] (Fig 1), *hCD2-iCre* mediated gene deletion had occurred in essentially all mature T and B cells (>96% EYFP^+^ cells, Fig 2). In contrast, macrophages and granulocytes showed negligible *hCD2-iCre* activity (<5% EYFP^+^ cells).

**Fig 3:**
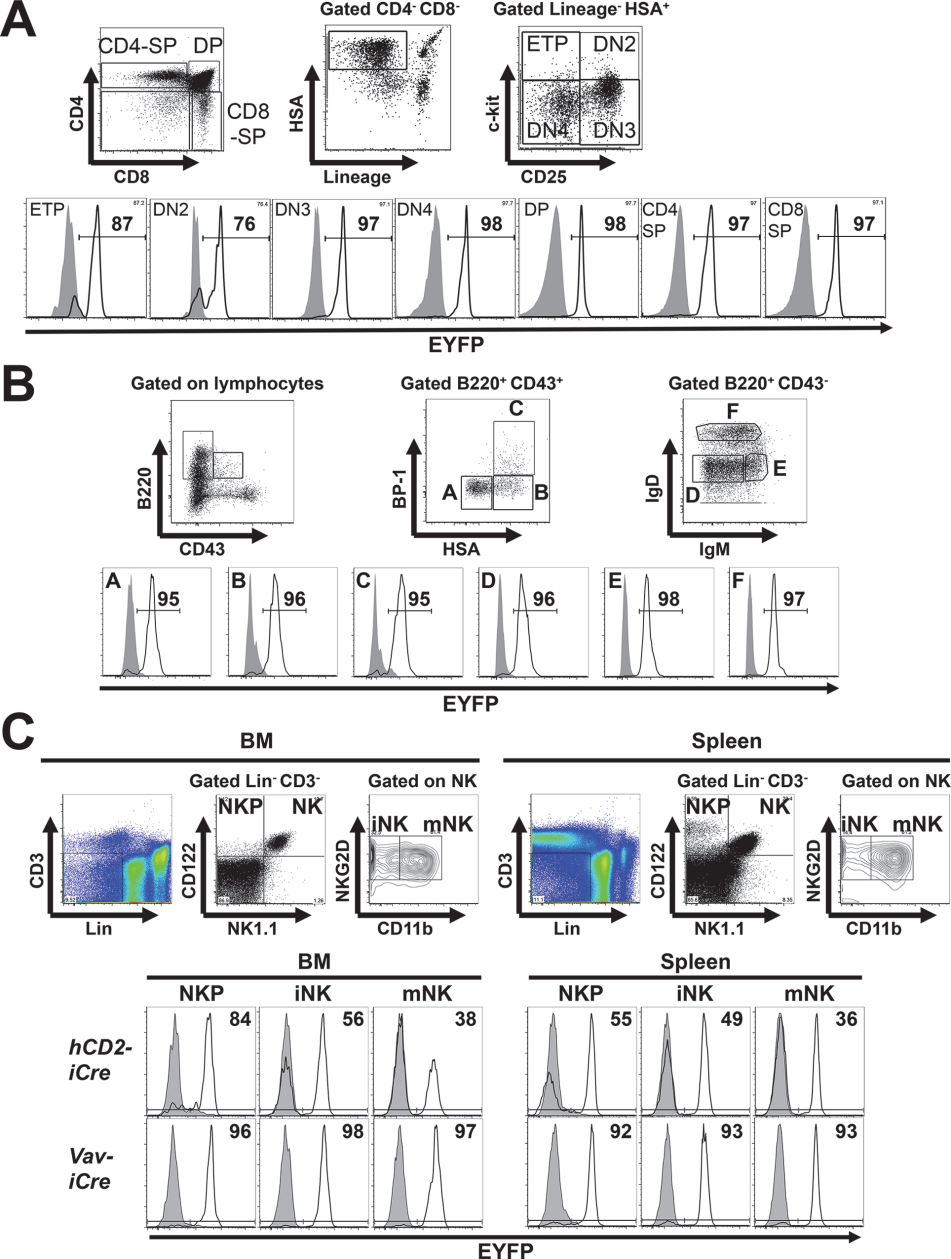
*hCD2-iCre* activity in T, B and NK cell development. **(A)** Thymocyte populations were identified by the gating strategy in the upper panel [13,31–33]. Lower panel, EYFP expression in the indicated thymocyte populations from *hCD2-iCre* ^*+/-*^ *R26-stop-EYFP* ^*+/-*^ (open histograms) or *R26-stop-EYFP* ^*+/-*^ mice (shaded histograms). Numbers denote % EYFP^+^ cells within the indicated population of *hCD2-iCre* ^*+/-*^ *R26-stop-EYFP* ^*+/-*^ mice. Representative of three independent experiments (n = 3). **(B)** Upper panel, subsets of developing B cells in the BM were distinguished as in [19]. Lower panel, EYFP expression in the indicated BM B cell populations from *hCD2-iCre* ^*+/-*^ *R26-stop-EYFP* ^*+/-*^ (open histograms) or *R26-stop-EYFP* ^*+/-*^ mice (shaded histograms). Numbers denote % EYFP^+^ cells within the indicated population of *hCD2-iCre* ^*+/-*^ *R26-stop-EYFP* ^*+/-*^ mice. Representative of three independent experiments (n = 3). **(C)** Upper panels, NK cell progenitors (NKP), immature (iNK) and mature (mNK) NK cells were identified using the indicated gating strategy [18,29]. Lower panels, EYFP expression in the indicated BM and splenic NK cell populations from (top, n = 3 per genotype) *hCD2-iCre* ^*+/-*^ *R26-stop-EYFP* ^*+/-*^ (open histograms) or *R26-stop-EYFP* ^*+/-*^ mice (shaded histograms), or (bottom, n = 2 per genotype) from *Vav-iCre* ^*+/-*^ *R26-stop-EYFP* ^*+/-*^ (open histograms) or *R26-stop-EYFP* ^*+/-*^ mice (shaded histograms). Numbers indicate % EYFP^+^ cells in the respective *Cre* ^*+/-*^ mice. Representative of three independent experiments with *hCD2-iCre* transgenic mice, and of two independent experiments with *Vav-iCre* transgenic mice.

Lymphoid NKT cells develop in the thymus from CD4^+^CD8^+^ precursors and have similar signaling requirements as developing T cells [28]. Both NKT precursors and NKT cells express *CD2* mRNA (Fig 1B and 1E). CD4^+^CD8^+^ thymocytes have high *hCD2-iCre* activity (Fig 3). Consistent with these findings, we found >83% EYFP^+^ NKT cells (Fig 2).

**Fig 4:**
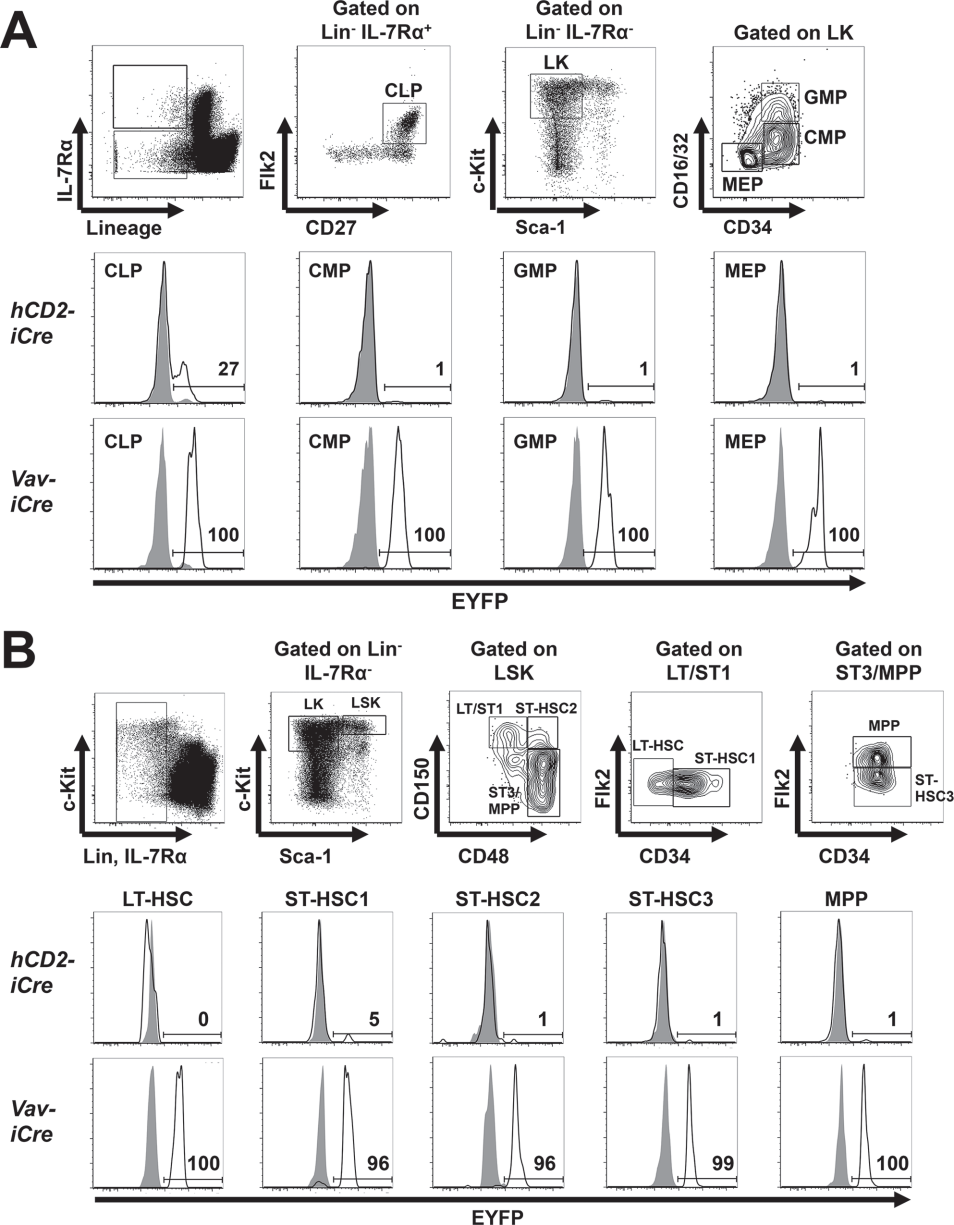
*hCD2-iCre* activity in HSC and HPC subsets. **(A)** Upper panels, HPC subsets were identified using the indicated gating strategy [24,25]. Lower panels, EYFP expression in CLP (Lin^-^Flk2^+^IL-7Rα^+^CD27^+^), MEP (Lin^-^IL-7Rα^-^c-kit^+^CD16/32^-^CD34^-^), CMP (Lin^-^IL-7Rα^-^c-kit^+^CD16/32^-/lo^CD34^+^) and GMP (Lin^-^IL-7Rα^-^c-kit^+^CD16/32^+^CD34^+^) from *hCD2-iCre* ^*+/-*^ *R26-stop-EYFP* ^*+/-*^ (open histograms) or *R26-stop-EYFP* ^*+/-*^ mice (shaded histograms), or from *Vav-iCre* ^*+/-*^ *R26-stop-EYFP* ^*+/-*^ (open histograms) or *R26-stop-EYFP* ^*+/-*^ mice (shaded histograms). Representative of three independent experiments. **(B)** Upper panels, phenotypic HSC/MPP subsets were identified using the indicated gating strategy [23,35,56]. Lower panels, EYFP expression in phenotypic LT-HSC (Lin^-^c-kit^+^Sca-1^+^CD150^+^CD34^-^CD48^-^Flk2^-^), ST-HSC1 (Lin^-^c-kit^+^Sca-1^+^CD150^+^CD34^+^CD48^-^Flk2^-^), ST-HSC2 (Lin^-^c-kit^+^Sca-1^+^CD150^+^CD34^+^CD48^+^Flk2^-^), ST-HSC3 (Lin^-^c-kit^+^Sca-1^+^CD150^-^CD34^+^CD48^+^Flk2^-^) and MPP (Lin^-^c-kit^+^Sca-1^+^CD150^-^CD34^+^CD48^+^Flk2^+^) from the mice in (A). Representative of at least two independent experiments. Numbers indicate % EYFP^+^ cells in the respective *Cre* ^*+/-*^ mice (n = 3 for experiments with *hCD2-iCre* transgenic mice, n = 2 for experiments with *Vav-iCre* transgenic mice).

Innate NK cells are commonly thought to develop from CLP via Lin^-^CD3^-^CD122^+^NK1.1^+^ NK cell progenitors (NKP) [14,18,29]. However, at least *in vitro*, human myeloid progenitors can also produce NK cells [30]. We found ≥36% EYFP^+^ mature NK1.1^+^CD11b^+^ splenic NK cells (mNK), ≥49% EYFP^+^ immature NK cells (iNK), ≥55% EYFP^+^ NK cell progenitors (NKP, Figs 2 and 3C) and ≥27% EYFP^+^ CLP (Fig 4A) in *hCD2-iCre* ^*+/-*^ *R26-stop-EYFP* ^*+/-*^ mice. This is consistent with *CD2* mRNA expression in NK cells (Fig 1A,1B and 1G) and supports derivation of many NKP and NK cells from CLP.

Dendritic cells include cDC with important roles in antigen presentation and T cell activation, and pDC capable of producing large type I IFN amounts upon viral encounter. CLP and common myeloid progenitors (CMP) can each give rise to cDC and pDC, although CLP contributions are thought to play a minor role under steady state conditions *in vivo*, in particular for cDC development [15–17]. Interestingly, we found 98% EYFP^+^ pDC but only ~20% EYFP^+^ cDC in *hCD2-iCre* ^*+/-*^ *R26-stop-EYFP* ^*+/-*^ mice (Fig 2B). Thus, although most cDC and pDC express no or very low *CD2* mRNA (Fig 1A,1F and 1G), *hCD2-iCre* is active in pDC and some cDC, or in their progenitors.

Altogether, our data indicate that *hCD2-iCre* leads to gene recombination not only in B and T cells, but also in iNKT cells, NK cells, pDC and some cDC.

### *hCD2-iCre* activity in T and B cell precursors

We next studied at which stage in T and B cell development *hCD2-iCre* mediated gene deletion can occur. T cell development occurs in the thymus and proceeds from Lin^-^HSA^high^CD4^-^CD8^-^CD44^+^CD25^-^c-Kit^+^ early thymocyte progenitors (ETP, a subset of DN1 cells) through successive CD44^+^CD25^+^c-Kit^+^ DN2, c-Kit^-^CD44^-^CD25^+^ DN3 and c-Kit^-^CD44^-^CD25^-^ DN4 stages followed by CD4^+^CD8^+^ double-positive (DP) and CD4^+^ or CD8^+^ single positive (SP) more mature stages [31–33]. Consistent with, and expanding previous reports [3,13], we detected robust EYFP expression in all these stages, including 87% EYFP^+^ ETP, 76% EYFP^+^ DN2 cells, and ≥97% EYFP^+^ DN3, DN4, DP and SP cells (Fig 3A). The higher proportion of EYFP^+^ ETP and DN2 cells in our study than previously found in DN1 and DN2 cells by others [3,13] likely reflects our more stringent gating on Lin^-^HSA^high^CD4^-^CD8^-^c-Kit^+^CD25^-^ (ETP) or Lin^-^HSA^high^CD4^-^CD8^-^c-Kit^+^CD25^+^ (DN2) cells, which defines both populations more purely [31–33]. As previously noted [13], the EYFP expression pattern is consistent with increasing *CD2* mRNA expression in post-DN3 stage thymocytes and mature T cells, but contrasts with the paucity of *CD2* mRNA in ETP and DN2 cells (Fig 1E and 1G).

B lymphocytes arise from CLP through a series of developmental stages in the BM [34]. A previous study reported *hCD2-iCre* induced EYFP expression in all peripheral B cells and BM-derived CD19^+^ IgD^-^ immature B cells [3], but the precise developmental stage where EYFP is first induced remained unclear. To better characterize *hCD2-iCre* activity during B cell development, we thus analyzed EYFP expression on successive stage A-C pro-B and early pre-B cells, stage D late pre-B cells, stage E newly formed/immature B cells and stage F recirculating mature/follicular B cells [19,34] in the BM of *hCD2-iCre* ^*+/-*^ *R26-stop-EYFP* ^*+/-*^ or *R26-stop-EYFP* ^*+/-*^ mice (Fig 3B). Interestingly, we found >95% EYFP^+^ cells in all B cell developmental subpopulations of *hCD2-iCre* ^*+/-*^ *R26-stop-EYFP* ^*+/-*^ but not control mice. This activity pattern is consistent with the high *CD2* mRNA expression in post-fraction C B cell developmental stages and mature B cells, but contrasts with the low *CD2* mRNA expression in fractions A-C (Fig 1A,1D and 1G).

Altogether, *hCD2-iCre* recombines *floxed* genes in all B and T cell developmental subsets, including the earliest thymic T cell and BM B cell precursors despite their low *CD2* mRNA expression levels.

### *hCD2-iCre* activity in early hematopoietic progenitor cell (HPC) and phenotypic HSC subsets

All blood cell lineages develop from pluripotent long-term repopulating hematopoietic stem cells (LT-HSC) via short-term repopulating ST-HSC, multipotent progenitors (MPP) and various HPC intermediates into mature cells [10,23,35,36]. MPP give rise to CLP and CMP. CLP generate pro-B cells and NKP which ultimately produce B and NK cells, although myeloid NK cell precursors might also exist [30]. CLP may also generate ETP and thus T cells and NKT cells, although the true nature of the thymus homing T cell progenitor is still under debate and HSC/MPP subsets may contribute to ETP generation without CLP involvement [37]. CMP generate granulocyte-macrophage progenitors (GMP) and megakaryocyte-erythrocyte progenitors (MEP), which then give rise to the myeloid and erythroid lineages, respectively. Both CLP and CMP can give rise to pDC and cDC subsets [15–17].

The high EYFP expression on *hCD2-iCre* ^*+/-*^ *R26-stop-EYFP* ^*+/-*^ ETP (Fig 3A) contrasts with the reported very low *CD2* expression in DN1 and DN2 cells (Fig 1E) and was hypothesized but not shown to reflect *CD2* expression in earlier progenitors [13]. Moreover, the EYFP expression in *hCD2-iCre* ^*+/-*^ *R26-stop-EYFP* ^*+/-*^ NKP, NK cells, pro-B cells, pDC and few cDC identified here (Figs 2 and 3) despite low *CD2* mRNA expression in pro-B cells and most DC (Fig 1A,1D,1F and 1G) would be consistent with *hCD2-iCre* activity in earlier hematopoietic progenitors. To test this possibility, we analyzed EYFP expression in BM HPC and phenotypic HSC subsets of *hCD2-iCre* ^*+/-*^ *R26-stop-EYFP* ^*+/-*^ and *R26-stop-EYFP* ^*+/-*^ mice (Fig 4A and 4B).

Consistent with essentially absent *CD2* mRNA expression (Fig 1C), phenotypic LT-HSC, ST-HSC2/3, MPP, CMP, GMP and MEP of *hCD2-iCre* ^*+/-*^ *R26-stop-EYFP* ^*+/-*^ mice were all negative for EYFP and thus lack *hCD2-iCre* activity (Fig 4A and 4B). The potential significance of a low 5% EYFP^+^ ST-HSC2 remains to be determined. In contrast, ~27% of *hCD2-iCre* ^*+/-*^ *R26-stop-EYFP* ^*+/-*^ CLP expressed EYFP (Fig 4A). Notably, CLP, ETP and fraction A B cell *CD2* mRNA levels are low but higher than in other HSC/HPC subsets (Fig 1C). This suggests that during hematopoietic development, *hCD2-iCre* mediated gene recombination starts in CLP and further increases in pro-B cells, ETP and NKP (Fig 3A–3C). The variable proportions of EYFP^+^ CLP might reflect the overall low *CD2* mRNA expression in CLP (Fig 1C), differences between individual mice or the relatively poorly defined CLP surface phenotype (Lin^-^ CD127^+^ Flk2^+^ CD27^+^) which might include contaminating EYFP^-^ non-CLP cells.

### CD45^-^ bone cells harbor *Vav-iCre* activity

For comparison with the *hCD2-iCre* ^*+/-*^ *R26-stop-EYFP* ^*+/-*^ mice, we analyzed EYFP expression in HSC and HPC of *Vav-iCre* ^*+/-*^ *R26-stop-EYFP* ^*+/-*^ and *R26-stop-EYFP* ^*+/-*^ mice. Consistent with previous reports [3,5–9], all HSC and HPC populations in *Vav-iCre* ^*+/-*^ *R26-stop-EYFP* ^*+/-*^ mice contained ≥96% EYFP^+^ cells (Fig 4A and 4B).

**Fig 5:**
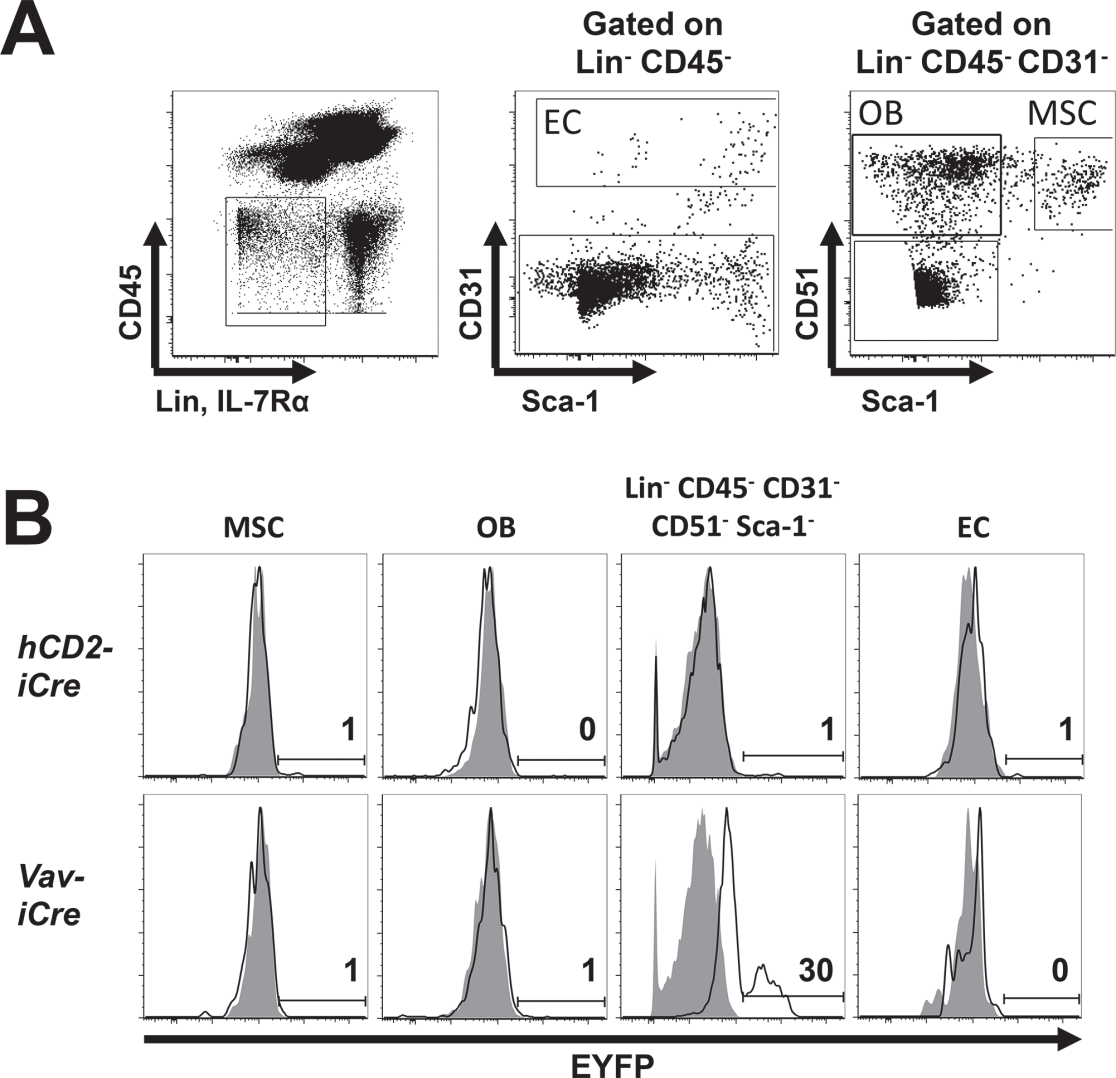
*Vav-iCre* activity in non-lymphoid bone cells. **(A)** Gating strategy. After flushing out the BM, bones were digested with collagenase and remaining cells stained for expression of the indicated markers. **(B)** EYFP expression on EC (Lin^-^CD45^-^CD31^+^), OB (Lin^-^CD45^-^CD31^-^CD51^+^Sca-1^-^), MSC (Lin^-^CD45^-^CD31^-^CD51^+^Sca-1^+^) and abundant Lin^-^CD45^-^CD31^-^CD51^-^Sca-1^-^ bone cells of unknown identity [22] from (upper panels) *hCD2-iCre* ^*+/-*^ *R26-stop-EYFP* ^*+/-*^ (open histograms) or *R26-stop-EYFP* ^*+/-*^ mice (shaded histograms), or from (lower panels) *Vav-iCre* ^*+/-*^ *R26-stop-EYFP* ^*+/-*^ (open histograms) or *R26-stop-EYFP* ^*+/-*^ mice (shaded histograms). Numbers indicate % EYFP^+^ cells in the respective *Cre* ^*+/-*^ mice. Representative of three independent experiments (n = 3).

Besides hematopoietic cells, older *Vav-Cre* lines also recombine *floxed* genes in the testis, and in vascular endothelial cells (EC) or precursors which do not express Vav, possibly due to *Cre-transgene* or *LacZ reporter* insertion effects [3,6]. To assess whether this also occurs in our *hCD2-iCre* and *Vav-iCre* lines, we next analyzed EYFP expression in stromal and endothelial cells from collagenase-digested, BM depleted bones [22]. We found no EYFP expression in *hCD2-iCre* ^*+/-*^ *R26-stop-EYFP* ^*+/-*^ and *Vav-iCre* ^*+/-*^ *R26-stop-EYFP* ^*+/-*^ Lin^-^CD45^-^CD31^+^ EC, Lin^-^CD45^-^CD31^-^CD51^+^Sca-1^-^ osteoblasts (OB) and Lin^-^CD45^-^CD31^-^CD51^+^Sca-1^+^ mesenchymal stem cells (MSC, Fig 5). As previously reported [22], most Lin^-^CD45^-^ cells in collagenase-digested bones were CD31^-^CD51^-^Sca-1^-^. About 30% of these cells were EYFP^+^, the other 70% EYFP^low^ in *Vav-iCre* ^*+/-*^ *R26-stop-EYFP* ^*+/-*^ but not *hCD2-iCre* ^*+/-*^ *R26-stop-EYFP* ^*+/-*^ mice (Fig 5), indicating a specific *Vav-iCre* activity. These cells are not EC and do not bear surface markers of mast cells (FcεR) or erythroid progenitors (CD41) as assessed by FACS. Their identity requires further investigation. Altogether, these data confirm potent *Vav-iCre* activity in all HSC and HPC subsets and identify a novel *Vav-iCre* activity containing Lin^-^CD45^-^CD31^-^CD51^-^Sca-1^-^ subset of bone cells.

## Discussion

Among several *Cre*-transgenes that allow conditional disruption of *floxed* genes in hematopoietic cells in mice, *Vav-iCre* is commonly used to recombine genes in HSC and all descendants, and *hCD2-iCre* for gene manipulation in B and T lymphocytes [2,3,12,13]. Here, we show that beyond their reported gene recombination patterns, *Vav-iCre* and *hCD2-iCre* also disrupt *floxed* genes in other murine cells. Besides hematopoietic cells, *Vav-iCre* recombined a *floxed EYFP* reporter gene also in a Lin^-^CD45^-^CD31^-^CD51^-^Sca-1^-^ bone cell type that lacks hematopoietic surface markers. Its identity remains to be elucidated. *hCD2-iCre* recombined a *floxed EYFP* reporter not only in B and T cells, but also in CLP, pro-B and all other stages of B cell development, ETP and subsequent stages of T cell development, NK cell progenitors, immature and mature NK cells, pDC and subsets of cDC.

Our analysis of *Vav-iCre* ^*+/-*^ *R26-stop-EYFP* ^*+/-*^ mice confirmed the previously reported *Vav-Cre* or *Vav-iCre* activity in all hematopoietic cell subsets [3,5–9]. In one other *Vav-Cre* transgenic mouse model, *Vav* was also expressed in germ cells and endothelial cells (EC) [6]. Similarly, the *Vav-iCre* transgene used in our study was previously reported to express *Vav* in testis and ovaries [3]. But in contrast to the other *Vav-Cre* line, our *Vav-iCre* transgenic mice showed no reporter gene recombination in vascular EC. We also found no *Vav-iCre* activity in osteoblasts and MSC (Fig 5), alleviating concerns that *Vav-iCre* activity in vascular niche cells could indirectly impact early hematopoiesis in the BM. However, we observed an unexpected *Vav-iCre* mediated EYFP expression in a Lin^-^CD45^-^CD31^-^CD51^-^Sca-1^-^ subset of bone cells. Although these cells have been observed elsewhere [22], their identity is unknown. *Vav-iCre* activity might suggest a hematopoietic origin, but their lack of hematopoietic markers (Fig 5) opens the possibility that these cells are non-hematopoietic, reminiscent of *Vav-iCre* expression in germ cells [3]. In the future, it will be important to determine whether these bone cells express endogenous Vav, and to elucidate their identity and function.

*hCD2-iCre* activity largely mirrors known patterns of *CD2* mRNA expression with the notable exception of CLP, pro-B cells, ETP and DN2 cells which all express relatively little *murine CD2* mRNA but display significant *hCD2-iCre* mediated EYFP expression. We speculate that this reflects a more efficient *hCD2-iCre* transgene expression combined with the high activity of the encoded improved Cre recombinase [3]. In any case, our data show that *hCD2-iCre* mediated gene recombination is essentially absent in HSC, MPP and myeloid progenitors, but initiates in CLP and affects early progenitors and all subsequent stages of the T cell, B cell and NK cell lineages, but no macrophages/monocytes and granulocytes. This recombination pattern strikingly resembles that of *Il7r* ^*Cre*^, where *iCre* was knocked into the *IL-7 receptor* locus and induced *flox-stop-flox ROSA26-YFP* expression in CLP, pro-T and T cells, B cells, NK cells, ~10% of cDC and >90% of pDC but not in other myeloid cells [38]. Similar to the *Il7r* ^*Cre*^ *R26-stop-YFP* ^*+*^ mice, the EYFP^+^ CLP in *hCD2-iCre* ^*+/-*^ *R26-stop-EYFP* ^*+/-*^ mice contained a large fraction of EYFP^low^ cells. This suggests that these CLP have only recently undergone Cre-mediated gene recombination, consistent with an initiation of Cre activity in CLP but not earlier progenitors. Supporting this view, *hCD2-iCre* only partially excised a *flox-stop-flox* cassette from a *TEL-AML1* fusion gene in CLP [39].

EYFP expression in CLP, ETP and all later stages of T cell development in both systems is consistent with a common lymphoid-primed origin for most T cells [13,38]. *hCD2-iCre* mediated EYFP expression in pro B cells and all later stages of B cell development, and in NKP, iNK and mNK moreover supports previously proposed common lymphoid origins of B cells and NK cells [14,40]. The existence of EYFP^-^ ETP in *hCD2-iCre* ^*+/-*^ *R26-stop-EYFP* ^*+/-*^ mice could reflect insufficient transgene activation in the specific progenitors that gave rise to these cells, or the existence of distinct *hCD2*-activating and non-activating T cell progenitor subsets, reminiscent of the hypothesized origin of *Il7r*-reporter activating and non-activating pro T cells from distinct BM progenitors whose identity remains to be determined [38]. Maximal *hCD2-iCre* activity in NKP with progressive reduction in iNK and mNK might indicate partial Cre-inactivation and outgrowth of undeleting cells after the NKP stage, or simply EYFP loss during red blood cell lysis as seen in T cells [3]. In an alternative possibility, it will be interesting to study if the emergence of *EYFP* ^*-*^ mature NK cells in *hCD2-iCre* ^*+/-*^ *R26-stop-EYFP* ^*+/-*^ mice, which was also seen in another study [41], ascribes *in vivo* relevance to the ability of myeloid progenitors to produce NK cells in human *in vitro* systems [30].

One of our most interesting findings is that 98% of splenic pDC, and 20% of splenic cDC express EYFP in *hCD2-iCre* ^*+/-*^ *R26-stop-EYFP* ^*+/-*^ mice (Fig 2). The hematopoietic origin of DC has long been controversial, mainly due to the ability of both lymphoid and myeloid progenitors to generate DC *in vitro* and after transplantation into lymphopenic mice [17,42]. Recently, IL-7 fate mapping [38] and the identification of a CMP-derived macrophage/DC progenitor (MDP) [43] and a downstream common DC precursor (CDP) capable of producing both pDC and cDC [44–46] have suggested a primarily myeloid origin in particular for cDC. On the other hand, lymphoid progenitor contributions would be consistent with the ability of CLPs to generate pDC and cDC, with the expression of Rag1 and detection of D-J rearranged *IgH* genes in splenic pDC [16,47–49], and with cell-intrinsic requirements for IL-7 signaling for the development of subsets of splenic pDC and cDC [50]. The EYFP reporter expression pattern on *hCD2-iCre* ^*+/-*^ *R26-stop-EYFP* ^*+/-*^ DC again strikingly resembles that on *Il7r* ^*Cre*^ *R26-stop-YFP* ^*+*^ DC [38] and might support the view that under steady-state *in vivo* conditions, many pDC and a minor fraction of cDC may develop from lymphoid progenitors. Reminiscent of the situation in ETP, *hCD2-iCre* induced EYFP expression in pDC and cDC despite their low *CD2* mRNA content (Fig 1A,1F and 1G) might possibly be explained by EYFP induction in upstream CLP (Fig 4). To further elucidate the origin of the EYFP expression in cDC from *hCD2-iCre* ^*+/-*^ *R26-stop-EYFP* ^*+/-*^ mice, it will be interesting to study if these cells emerge from EYFP^+^ CD11c^+^B220^+^CCR9^-^ cDC progenitors [51]. These are included in our pDC gate, which contains 98% EYFP^+^ cells. In any case, our data are consistent with contributions of both myeloid and lymphoid progenitors to pDC and cDC development [17,42]. They indicate that contributions of altered DC function to any phenotypes of *hCD2-iCre* based conditional knockout mice need to be considered, in particular when studying T cells which are activated by antigens presented on DC.

Again similar to *Il7r* ^*Cre*^ *R26-stop-YFP* ^*+*^ mice [38], we found low-level (<5%) EYFP expression in *hCD2-iCre* ^*+/-*^ *R26-stop-EYFP* ^*+/-*^ macrophages and granulocytes (Fig 2) but not CMP, GMP and MEP (Fig 4). Although these EYFP^+^ myeloid cells are very rare, they could reflect the ability of CLP and T lineage precursors to generate myeloid cells under appropriate conditions, or the ability of certain neutrophil subsets to express lymphoid markers, discussed in detail elsewhere [38].

Altogether, our data confirm and expand the EYFP expression in developing and mature T cells, B cells and NK cells previously found in the same *hCD2-iCre* transgenic line [3,41] and the EYFP expression in developing and mature T cells and B cells in other *CD2-Cre* transgenics [3,12,13]. One study also reported mosaic EYFP expression in the testis of *hCD2-iCre* transgenic males but not in ovaries in females [3]. A *hCD2* transgenic mouse line expressed hCD2 protein in T cells, but not in B cells [52]. In mice harboring multiple *hCD2* transgene copies, immunohistochemistry suggested hCD2 protein expression in T cells, megakaryocytes, platelets and bone marrow cells of unknown identity with convoluted nuclei speculated to be myeloid precursors [52]. This contrasts with the lack of EYFP expression in myeloid progenitors and MEP in our mice (Fig 4) but could be consistent with the low level EYFP expression we found in macrophages and granulocytes (Fig 2), and with myeloid contributions to the EYFP^+^ DC in our mice (Fig 2).

The differences in transgene expression between different mouse lines in part result from differences in the precise *Vav* or *hCD2* gene regions and supporting features included in each transgene. Moreover, during the generation of transgenic mice, the transgene integrates randomly and in variable copy numbers into the genome. Chromatin context affects transgene expression levels. During breeding, meiotic recombination might affect transgene structure and copy numbers. This, differential maternal/paternal effects and differences in genetic backgrounds such as the C57BL/6-J129 mixed background of the *R26-stop-EYFP* ^*+/-*^ mice can lead to variability in transgene expression levels between different mouse lines and even between individual mice of the same line [4]. These unavoidable limitations of transgenic mice likely explain the differences between our data and previously published data even using the same *Cre*-transgenic lines.

This notwithstanding, our data provide important novel insight into the *Vav-iCre* and *hCD2-iCre* transgene activity patterns in hematopoietic and non-hematopoietic cell populations. They suggest applications for these transgenes for conditional gene modulation in novel cell types, and provide a map of cell types whose extrinsic contributions to phenotypes of *Vav-iCre* or *hCD2-iCre* mediated gene modulation in a cell type of interest must be taken into account when interpreting results.

Finally, we propose that in addition to *Il7r* ^*Cre*^ *R26-stop-YFP* ^*+*^ mice [38], *hCD2-iCre* ^*+*^ *R26-stop-EYFP* ^*+*^ mice are an excellent genetic tool for lineage tracing studies of lymphoid cells *in vivo* that avoids the often non-physiological differentiation potential of CLP or other lymphoid precursors *in vitro* or upon engraftment into lymphopenic hosts [38].
